# Educational attainment in poor comprehenders

**DOI:** 10.3389/fpsyg.2014.00445

**Published:** 2014-05-28

**Authors:** Jessie Ricketts, Rachael Sperring, Kate Nation

**Affiliations:** ^1^Institute of Education, University of ReadingReading, UK; ^2^Department of Experimental Psychology, University of OxfordOxford, UK

**Keywords:** poor comprehenders, educational attainment, reading comprehension, specific reading comprehension impairment, oral vocabulary

## Abstract

To date, only one study has investigated educational attainment in poor (reading) comprehenders, providing evidence of poor performance on national UK school tests at age 11 years relative to peers (Cain and Oakhill, 2006). In the present study, we adopted a longitudinal approach, tracking attainment on such tests from 11 years to the end of compulsory schooling in the UK (age 16 years). We aimed to investigate the proposal that educational weaknesses (defined as poor performance on national assessments) might become more pronounced over time, as the curriculum places increasing demands on reading comprehension. Participants comprised 15 poor comprehenders and 15 controls; groups were matched for chronological age, nonverbal reasoning ability and decoding skill. Children were identified at age 9 years using standardized measures of nonverbal reasoning, decoding and reading comprehension. These measures, along with a measure of oral vocabulary knowledge, were repeated at age 11 years. Data on educational attainment were collected from all participants (*n* = 30) at age 11 and from a subgroup (*n* = 21) at 16 years. Compared to controls, educational attainment in poor comprehenders was lower at ages 11 and 16 years, an effect that was significant at 11 years. When poor comprehenders were compared to national performance levels, they showed significantly lower performance at both time points. Low educational attainment was not evident for all poor comprehenders. Nonetheless, our findings point to a link between reading comprehension difficulties in mid to late childhood and poor educational outcomes at ages 11 and 16 years. At these ages, pupils in the UK are making key transitions: they move from primary to secondary schools at 11, and out of compulsory schooling at 16.

## INTRODUCTION

In the early stages of learning to read, children must learn to map letters onto sounds so that they can decode and recognize words. However, the ultimate goal of reading is to understand the messages conveyed by text; simply being able to read words and texts accurately is not sufficient for comprehension to occur. A substantial number of children (~8% in UK studies; Clarke et al., 2010) show reading comprehension impairments despite age-appropriate word recognition skills; these children are typically referred to as “poor comprehenders” or “children with specific reading comprehension impairments.” Research conducted in Italy, the UK and the US has made good progress with understanding the cognitive and linguistic profiles that characterize poor comprehenders in mid to late childhood (e.g., poor oral language, poor inferential skills; for reviews, see Nation, 2005; Floyd et al., 2006; Cain and Oakhill, 2007; Carretti et al., 2009) but we know very little about the progress that such children make in adolescence, and at school. We conducted a longitudinal study tracking reading, vocabulary, and educational attainment in poor comprehenders over the course of eight years: from age 9 to 16 years. Educational attainment was indexed through performance on national UK school assessments at the end of primary school (11 years) and at the end of compulsory education (16 years)^1^. Given that poor comprehenders struggle to learn from what they read (Cain et al., 2004; Ricketts et al., 2008), and that acquiring knowledge through the process of reading becomes an increasingly important learning strategy as children move through the school system, it seems likely that poor comprehenders will be at a disadvantage at school. Despite the likely educational consequences of the reading comprehension difficulties experienced by poor comprehenders, their difficulties may be masked by good reading accuracy in the classroom (Nation and Angell, 2006; Hulme and Snowling, 2011), and only one study to date has investigated educational attainment in this group (Cain and Oakhill, 2006).

Research with poor comprehenders has shed light on the factors, beyond word recognition, that support successful reading comprehension, particularly focussing on oral language (e.g., Catts et al., 2006; Nation et al., 2010), discourse level processes such as inference generation and comprehension monitoring (e.g., Oakhill and Cain, 2012) and executive functions such as working memory (e.g., Carretti et al., 2009). Longitudinal data and intervention studies provide particularly convincing evidence for causal relationships. However, there is a dearth of longitudinal and intervention research with poor comprehenders. Nonetheless, existing longitudinal studies indicate that poor oral language can be observed in poor comprehenders before their reading comprehension difficulties are identified, suggesting that oral language weaknesses precede (and therefore may cause) their reading comprehension difficulties. In a US study, Catts et al. (2006) selected 57 poor comprehenders in eighth Grade (14 years) and looked retrospectively at their oral language skills in Kindergarten, second Grade and fourth Grade (age 6, 8, and 10 years, respectively). Poor comprehenders performed more poorly than typically developing readers on a language comprehension composite at each time point. In the UK, Nation et al. (2010) conducted a prospective longitudinal study, assessing oral language and reading in 242 children for the first time at age 5 years and following children over time until poor comprehenders (*n *= 15) could be reliably identified at age 8 years. Again, weaknesses in oral language comprehension were detected earlier in time, when children had experienced very little reading instruction. In the only randomized controlled trial conducted with poor comprehenders to date, Clarke et al. (2010) showed significant improvements in reading comprehension scores following an oral language intervention program, concluding that oral language weaknesses play a causal role in determining the reading comprehension difficulties that are experienced by poor comprehenders (aged 8–9 years). At present however, we know very little about poor comprehenders later in development, as they transition to secondary school and beyond.

The idea that oral language skills such as vocabulary and grammar provide a foundation for successful reading comprehension is embodied by the Simple View of Reading (Gough and Tunmer, 1986; Tunmer and Chapman, 2012), a key theoretical framework that has been used to conceptualize reading development and reading difficulties. On this view, word recognition and oral language comprehension are separable variables that underpin reading comprehension, and both are necessary for successful reading (reading for meaning). Substantial support for the assumptions of the Simple View derive from a wide range of empirical approaches, including longitudinal research with typically developing children (e.g., Oakhill et al., 2003; Muter et al., 2004), the study of children with specific reading difficulties (e.g., Catts et al., 2006), behavioral genetics (e.g., Harlaar et al., 2010) and factor analysis (e.g., Tunmer and Chapman, 2012). Despite its wide use in reading research, the Simple View is not without its critics. Notable are arguments that the word recognition and oral language comprehension components of the Simple View are poorly specified, that they are not entirely independent, and that reading comprehension involves more than just these components (e.g., Kirby and Savage, 2008; Ouellette and Beers, 2010; Tunmer and Chapman, 2012; Ricketts et al., 2013).

The relationship between oral language and reading is reciprocal, with reading activities providing important opportunities for growth in aspects of oral language such as vocabulary knowledge (e.g., Nagy et al., 1985). Importantly, the extent to which children learn new words while reading will depend on their reading proficiency (e.g., Ricketts et al., 2011). Poor comprehenders show particular difficulty learning and retaining the meanings of novel words from context (Cain et al., 2003, 2004; Ricketts et al., 2008), suggesting that slowed growth in vocabulary (Matthew effects) is a possibility in this group. As mentioned above, few studies have tracked development in poor comprehenders (for a summary of existing studies, see Elwér et al., 2013). Nonetheless, the longitudinal work of Cain and Oakhill (2011) lends support to the hypothesis that poor comprehenders show Matthew effects for vocabulary. Matthew effects refer to the widening of gaps between low and high achievers over time (Stanovich, 1986). Cain and Oakhill (2011) assessed reading and receptive vocabulary in 17 poor comprehenders and 14 good comprehenders at ages 8 and 11 years. Using Scarborough and Parker’s (2003) ANOVA approach for detecting Matthew effects, Cain and Oakhill (2011) demonstrated slowed receptive vocabulary growth in poor relative to good comprehenders. In contrast, differences between groups were relatively constant over time for reading comprehension, indicating persistent reading comprehension impairments in the poor comprehenders (see also Cain and Oakhill, 2006).

Reading for meaning provides not only important opportunities for the acquisition of vocabulary and other aspects of language, but also for learning more generally. As mentioned above, it is likely that reading comprehension impairments will be associated with poor educational outcomes and yet only one UK-based study has explored educational attainment in poor comprehenders. In the UK, children complete national School Assessment Tests (SAT-UK) tests at 11 years, just before they transition from primary to secondary school. Currently, SAT-UK tests focus on English and maths curriculum subjects, but in the past science was also examined. Cain and Oakhill (2006) reported data from SAT-UK tests for 16 poor comprehenders and 17 good comprehenders who had been identified 3 years earlier (age 8 years) from UK primary schools. Cain and Oakhill (2011) found that group means for poor and good comprehender groups were in line with government targets (a level 4). However, the good comprehender group obtained a significantly higher mean score than the poor comprehender group on English, maths, and science SAT-UK tests. Thus, Cain and Oakhill’s (2011) study indicates that, on average, poor comprehenders attain at an age-appropriate level at age 11 years. However, they are at a disadvantage in comparison to peers without a history of reading comprehension difficulty.

The primary aim of the present study was to investigate educational attainment in poor comprehenders. To this aim, we collected longitudinal data over a period of 8 years, identifying poor comprehenders and age-matched controls without reading comprehension difficulties at age 9 years, and recording their performance in national UK school assessments at the end of primary school (SAT-UK tests at 11 years) and at the end of compulsory education (16 years). At 16 years, pupils in the UK sit General Certificate of Secondary Education (GCSE) tests and equivalents; the present study investigates GCSE attainment in poor comprehenders for the first time (for studies on GCSE performance of children with a history of primary language impairment, see Snowling et al., 2001; Dockrell et al., 2011). Both SAT-UK and GCSEs are described in more detail later in this paper. Based on Cain and Oakhill (2006), we anticipated that as a group, poor comprehenders’ SAT-UK attainment would be in line with national norms but that poor comprehenders would perform more poorly than controls.

We sought to build on Cain and Oakhill’s (2006) study in two ways. First, Cain and Oakhill (2011) did not report individual scores on SAT-UK tests. Given the heterogeneous nature of poor comprehender groups (Nation et al., 2002; Cain and Oakhill, 2006; Floyd et al., 2006), we sought to examine individual profiles to ascertain whether there are poor comprehenders who are attaining below national expectations as they transition from the primary school curriculum to its more demanding secondary counterpart. Second, we collected data on national assessments at the end of compulsory schooling in the UK to investigate longer term educational outcomes for children who had been identified as poor comprehenders in middle childhood. We anticipated that as the curriculum places greater demands on reading comprehension, group differences in attainment might become more pronounced and that later in the educational system poor comprehenders might show evidence of falling behind government targets.

Measures of reading comprehension and expressive oral vocabulary were administered at ages 9 and 11 years. Therefore, in addition to exploring educational progress, we sought to replicate studies showing that the reading comprehension difficulties experienced by poor comprehenders are persistent over time (Cain and Oakhill, 2006, 2011) and to investigate oral vocabulary development in this group. Given evidence for poor vocabulary learning (Cain et al., 2003, 2004; Nation et al., 2007; Ricketts et al., 2008) and slowed receptive vocabulary development in poor comprehenders (Cain and Oakhill, 2011), we expected to see Matthew effects for vocabulary.

To our knowledge, the present study is the first of its kind, tracking development in poor comprehenders over a particularly long timeframe: from identification at age 9 years to adolescence (16 years). By considering reading and vocabulary at 9 years (Time 1) and 11 years (Time 2), and attainment as measured by UK national school assessments at 11 years (Time 2) and 16 years (Time 3), we sought to address the following key research questions:

1. Do poor comprehenders show low educational attainment at ages 11 and 16 years compared to controls (typically developing readers) matched for age, nonverbal reasoning, and decoding (nonword reading)?

2. Do poor comprehenders show poor educational attainment at ages 11 and 16 years as compared to the attainment of pupils nationally in the UK?

3. Are the reading comprehension difficulties experienced by poor comprehenders stable over time?

4. Do poor comprehenders show Matthew effects for vocabulary (i.e., slowed growth)?

## MATERIALS AND METHODS

### PARTICIPANTS

Participants were 15 poor comprehenders and 15 controls drawn from a sample of 81 children who were attending mainstream schools that serve socially mixed catchment areas in the UK. None of the larger sample of 81 children spoke English as an additional language or had any recognized special educational need. Participants for each group were selected according to the following criteria. Poor comprehenders obtained reading comprehension standard scores of at least one standard deviation below the test mean (≤85) and controls’ scores were well into the average range or above (>95). Groups were matched for chronological age, nonverbal reasoning ability and decoding (nonword reading) skill, with all children performing within the average range (or above) on nonverbal reasoning and decoding tasks. Groups were also matched for gender, with 11 girls and 4 boys in each group. Details of all measures are included below and performance of both groups is summarized in **Table 1**. Ethical approval for the study was obtained from the University of Oxford (Time 1 and Time 2) and University of Reading (Time 3) Research Ethics Committees.

**Table 1:** Summary of performance on selection measures, follow up measures and oral vocabulary at Time 1 and Time 2.

Time point/measure	Poor comprehenders (*n* = 15)		Controls (*n* = 15)		Group comparisons		
	*M*	*SD*	*M*	*SD*	*F*	*p*	Cohen’s *d*
**Time 1 selection**
Chronological age^1^	9.21	0.30	9.26	0.28	0.22	0.64	0.17
Nonverbal reasoning^2^	103.50	7.54	103.50	6.43	0.00	1.00	0.00
Decoding^2^	107.67	13.11	108.27	9.68	0.02	0.88	0.05
Reading comprehension^2^	81.93	2.69	103.13	4.88	217.14	<0.001	5.60
**Time 2 follow up**
Chronological age^1^	11.30	0.31	11.34	0.32	0.14	0.71	0.12
Nonverbal reasoning^2^	101.90	6.98	103.50	8.63	0.31	0.58	0.20
Decoding^2^	106.40	14.94	107.27	10.66	0.03	0.86	0.07
Reading comprehension^2^	83.60	4.44	95.87	7.57	29.33	<0.001	2.04
**Oral vocabulary**
Time 1^2^	89.20	13.63	110.00	8.09	25.82	<0.001	1.92
Time 2^2^	91.50	11.31	109.50	6.12	29.40	<0.001	2.07

*^1^Years; ^2^Standard scores (*M *= 100, *SD* = 15).*

### MATERIALS AND PROCEDURE

Poor comprehenders and controls were identified at Time 1 using the standardized measures of nonverbal reasoning, decoding, and reading comprehension outlined below. These measures, along with a measure of oral vocabulary knowledge, were repeated at Time 2, approximately 2 years later (*M *time difference = 2.08 years, *SD *= 0.12, range: 1.83–2.29). Note that participants completed other tasks in between these two testing points, which are reported elsewhere (Ricketts et al., 2007, 2008). All standardized measures were administered according to manual instructions. Data on educational attainment were collected at the end of primary school (Time 2) and approximately 5 years later at the end of compulsory education (Time 3).

#### Nonverbal reasoning

Nonverbal reasoning was measured using the Matrix Reasoning subtest of the Wechsler Abbreviated Scale of Intelligence (WASI; Wechsler, 1999). This subtest assesses nonverbal reasoning using a pattern completion task in which participants are provided with a pattern that has a piece missing; their task is to select the missing piece from an array of five. WASI subtests yield a *t*-score (*M *= 50, *SD* = 10); for comparison with other measures, this was transformed into a standard score (*M *= 100, *SD* = 15). The WASI provides norms for individuals aged 6–89 years, and high internal consistency (split half reliability) is reported in the manual (*r* = 0.86–0.96, depending on age group).

#### Oral vocabulary

Oral vocabulary knowledge was measured using the Vocabulary subtest of the WASI (Wechsler, 1999). This is a measure of expressive vocabulary in which children are asked to verbally define words. Scores capture both depth and breadth of word knowledge, indexing the incremental nature of oral vocabulary knowledge. WASI subtests yield a *t*-score (*M *= 50, *SD* = 10); for comparison with other measures, this was transformed into a standard score (*M *= 100, *SD* = 15). The WASI provides norms for individuals aged 6–89 years, and high internal consistency (split half reliability) is reported in the manual (*r* = 0.86–0.93, depending on age group).

#### Decoding

Decoding (nonword reading) was assessed using the phonemic decoding efficiency (PDE) subtest of the test of word reading efficiency (TOWRE; Torgesen et al., 1999). In this test, children are asked to read a list of nonwords of increasing length and difficulty as quickly as they can. Efficiency is indexed by the number of nonwords decoded correctly in 45 s. The TOWRE produces standard scores (*M *= 100, *SD* = 15). The test provides norms for individuals aged 6–24 years, and its manual indicates a high level of test/re-test reliability (*r* = 0.89–0.91, depending on age group).

#### Reading comprehension

Reading comprehension was assessed using the Neale Analysis of Reading Ability-II (NARA-II; Neale, 1997). In the NARA-II children read aloud passages of connected text and then answer comprehension questions relating to each passage. Some questions can be answered with reference to verbatim memory while others require inferences to be made (Bowyer-Crane and Snowling, 2005). The NARA-II comprises two parallel forms; children completed Form 1 at Time 1 and Form 2 at Time 2 to avoid practice effects. The NARA-II produces standard scores (*M *= 100, *SD* = 15) for reading comprehension. The test provides norms for children aged 6–12 years, and shows high internal consistency (Cronbach’s α**= 0.93–0.95, depending on age group). The manual reports high correlations between comprehension scores on the two parallel forms (*r *= 0.82).

#### Educational attainment

In England, pupils sit national school assessments at the end of primary school at age 11 years (SAT-UK tests) and at the end of compulsory education at age 16 years (GCSEs or qualifications at an equivalent level). At Time 2, participants were in the final year of primary school and at the end of this year schools were contacted to obtain SAT-UK test results. Schools provided the level (from 2 to 5) at which all pupils (*n *= 30) were performing in English, maths, and science subjects (note that pupils no longer sit SAT-UK tests for science). English results can be further decomposed into separate scores for reading and writing. Given the reading difficulties observed in the poor comprehenders, reading and writing scores were considered separately. Maths and science scores were considered to aid comparison with an earlier study (Cain and Oakhill, 2006). UK government targets stipulate that in order to be “secondary ready” (have the requisite knowledge and skill to manage the secondary curriculum) pupils should be operating at level 4 or above at the end of primary school. The UK government publishes data each year indicating how many children meet this target (UK Department for Education, 2012a). Not all pupils obtain a level 4 in each subject but the majority do; thus, a level 4 does not represent the average, instead, most children are expected to reach this level.

At Time 3, GCSE (or equivalent) results were obtained via the following process. Some primary schools provided information about secondary school destinations at Time 2. For the remaining participants, primary schools were contacted and asked to provide details of secondary school destinations. The secondary schools that consented to take part in the study distributed information sheets and consent forms to participants and, on the basis of informed consent, released GCSE results to the research team. This process yielded GCSE data for 20/30 participants. One secondary school and one participant did not consent to take part. For some of the remaining participants, home addresses had been provided by parents at Time 1 (but this was not compulsory for inclusion in the study). Where possible, participants for whom GCSE data had not been obtained from schools were contacted directly by post. This resulted in one participant sending information about GCSE results independently. Thus, GCSE results were available for 21/30 participants.

GCSE-level qualifications can be acquired for a wide range of curriculum subjects, including the SAT-UK subjects (English, maths, science) as well as other subjects (e.g., foreign languages, history, geography, art). Pupils and schools work together to choose the number of qualifications a pupil undertakes and which subjects they study at this level. When GCSEs (or equivalents) are marked, grades are given (A*, A, B–G) that correspond to points (16–58, e.g., A* = 58, *A* = 52, *B* = 46, *C* = 40). Grades fall into two levels, level 2 relates to grades A^*^–C, and level 1 to grades D–G. Grades and points determine, to some extent, post-16 destinations (further education, apprenticeships, employment opportunities, etc.). When the government reports on attainment for pupils in England at the end of compulsory education, two key variables of interest are whether children obtained five GCSEs (or equivalent) at level 2 (i.e., with grades between A* and C) and whether they have made “expected progress” since taking SAT-UK tests. Expected progress is only recorded for subjects taken at both SAT-UK level (now English, maths) and GCSE (English and maths are compulsory); within each subject this reflects a pupil obtaining a level 3, 4, or 5 at SAT-UK and then at least D, C, or B at GCSE, respectively. The UK government publishes data each year indicating how many children meet these targets (UK Department for Education, 2012b).

## RESULTS

### READING AND VOCABULARY AT TIME 1 AND TIME 2

**Table 1** summarizes age and performance (standard scores) on nonverbal reasoning, decoding, and reading comprehension measures at Time 1 (selection measures) and Time 2 as well as performance on an oral vocabulary measure at Time 1 and Time 2. **Table 1** also includes details of group comparisons (one-way ANOVA) for each variable. In line with selection and matching procedures, groups were closely matched for age, nonverbal reasoning and decoding at Time 1. This close correspondence between the two groups was maintained at Time 2. Groups differed on reading comprehension and oral vocabulary measures at Time 1 and Time 2, with large effect sizes observed (all Cohen’s *d *≥ 2).

**FIGURE 1:**
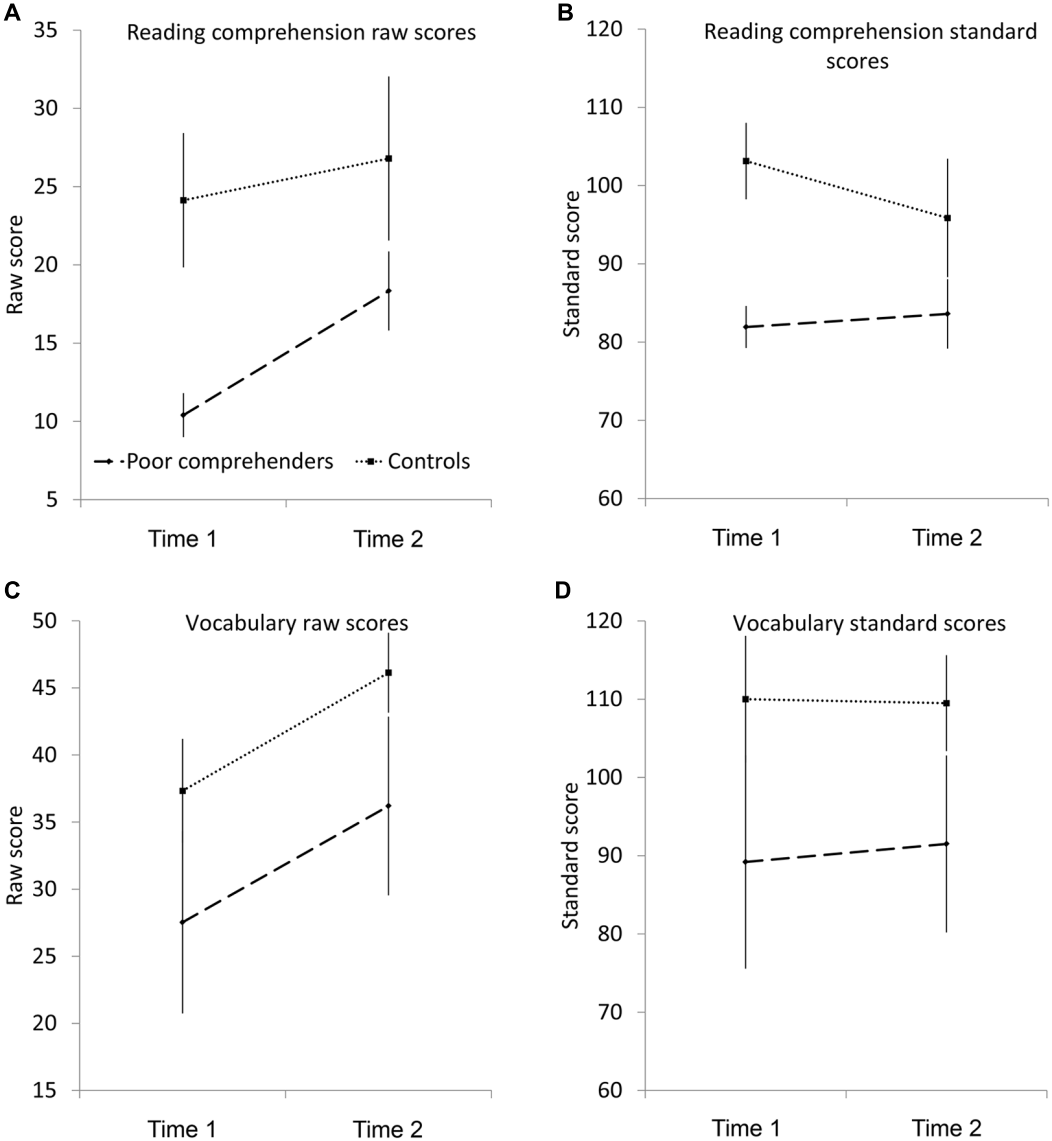
**Mean reading comprehension raw scores (A), reading comprehension standard scores (B), vocabulary raw scores (C), and vocabulary standard scores (D) for poor comprehenders (solid line) and controls (broken line) at Time 1 and Time 2**.

To investigate Matthew effects, data on reading comprehension and oral vocabulary were analyzed using a series of 2 × 2 ANOVAs; in each, group (poor comprehenders vs. controls) was included as an independent samples factor and time (Time 1 vs. Time 2) as a repeated samples factor. Both raw scores and standard scores for each variable (reading comprehension, vocabulary) were analyzed to probe changes in absolute score (number of comprehension questions correct, knowledge of vocabulary items) as well as norm-referenced scores (cf. Scarborough and Parker, 2003; Cain and Oakhill, 2011). Mean raw scores on reading comprehension and oral vocabulary tasks are depicted in **Figures 1A, C**, respectively; mean standard scores appear in **Table 1** but are replicated in **Figures 1B, D** for ease of comparison.

When reading comprehension raw score (max = 44) was the dependent variable (**Figure 1A**), the main effect of group was significant, *F*(1,28) = 86.98, *p *< 0.001, $\eta_{p}^{2}$ = 0.76, with controls outperforming poor comprehenders, as was the main effect of time, *F*(1,28) = 71.88, *p *< 0.001, $\eta_{p}^{2}$ = 0.72, with higher performance at Time 2. These main effects were qualified by a significant group x time interaction, *F*(1,28) = 17.75, *p *< 0.001, $\eta_{p}^{2}$ = 0.39. Tests of simple effects with Bonferroni correction revealed that both groups showed a significant increase in raw score over time, but the poor comprehender group showed greater improvement. There were significant group differences in raw score at both time points but this was more marked at Time 1. When reading comprehension standard score was the dependent variable (**Figure 1B**) the main effects of group, *F*(1,28) = 105.01, *p *< 0.001, $\eta_{p}^{2}$ = 0.79, and time, *F*(1,28) = 8.40, *p *< 0.01, $\eta_{p}^{2}$ = 0.23, were also significant. Again, main effects were qualified by a significant group × time interaction, *F*(1,28) = 21.37, *p *< 0.001, $\eta_{p}^{2}$ = 0.43. Tests of simple effects with Bonferroni correction revealed that for the control group there was a significant decrease in the mean reading comprehension standard scores between Time 1 and Time 2, indicating that for this group reading comprehension performance was not developing in line with cross-sectional data from the test’s normative sample. As would be expected from the test norms, means were stable across time (did not change significantly) for the poor comprehender group.

**FIGURE 2:**
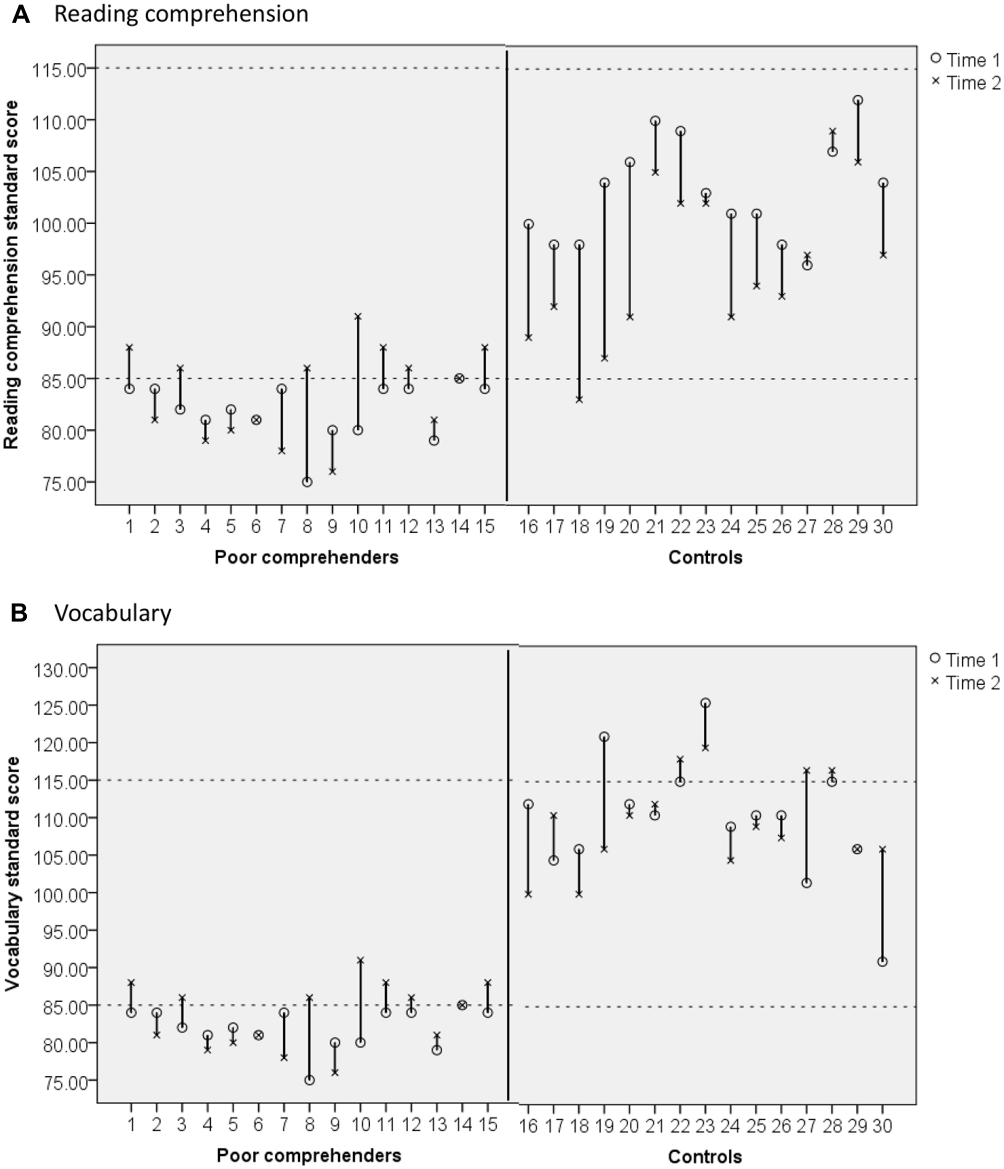
**Change in (A) reading comprehension standard scores and (B) oral vocabulary standard scores between Time 1 (circles) and Time 2 (crosses) in each participating poor comprehender (participants 1–15) and control (participants 16–30).** Participant numbers are equivalent across **(A,B)** such that participant 1 in **(A)** is the sample child as participant 1 in **(B)**.

In line with our aim to consider development at the individual level, changes in individual reading comprehension scores are depicted in **Figure 2A** for reference. At Time 2, eight of the 15 poor comprehenders (53%) obtained reading comprehension standard scores that were at least one standard deviation below the test mean; all of these children still met the strict identification criteria adopted at Time 1 (see above). The remaining seven poor comprehenders obtained reading comprehension standard scores that were slightly greater than 85. At Time 2, most poor comprehenders still showed the large discrepancy between advanced decoding and lower reading comprehension that characterizes the poor comprehender profile (*M* discrepancy = 22.80, *SD* = 16.25). One participant in the control group also met poor comprehender criteria at Time 2.

**Figures 1C, D** shows mean oral vocabulary raw scores (max = 80) and standard scores for poor comprehenders and controls at Time 1 and Time 2. The 2 × 2 ANOVA with oral vocabulary raw score as the dependent variable revealed significant main effects of group, *F*(1,28) = 31.04, *p *< 0.001, $\eta_{p}^{2}$ = 0.53, and time, *F*(1,28) = 113.65, *p *< 0.001, $\eta_{p}^{2}$ = 0.80, with controls outperforming poor comprehenders and higher performance at Time 2 than Time 1. The group × time interaction was not significant, *F*(1,28) = 0.68, *p *= 0.42, $\eta_{p}^{2}$ = 0.02, consistent with the parallel lines in **Figure 1**. With oral vocabulary standard score as the dependent variable, again there was a significant main effect of group, *F*(1,28) = 34.14, *p *< 0.001, $\eta_{p}^{2}$ = 0.55, but the main effect time, *F*(1,28) = 0.28, *p *= 0.60, $\eta_{p}^{2}$ = 0.01, and group × time interaction, *F*(1,28) = 0.68, *p *= 0.42, $\eta_{p}^{2}$ = 0.02, were not significant. Changes in individual vocabulary scores are depicted in **Figure 2B**.

### EDUCATIONAL ATTAINMENT AT 11 YEARS (TIME 2)

**FIGURE 3:**
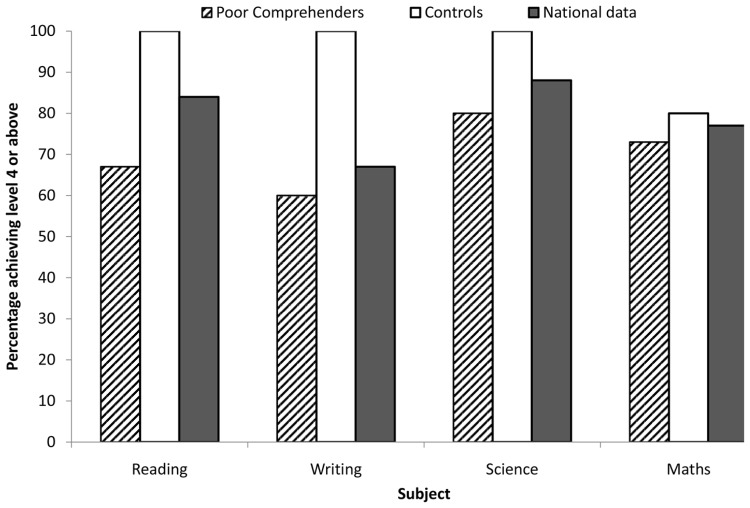
**Percentage of children in the poor comprehender group, control group, and nationally, achieving level 4 or above in reading, writing, science and maths SAT-UK tests at 11 years.** National data refer to children in England completing SAT-UK tests at the same time as poor comprehenders and controls.

Government targets stipulate that children should be performing at or above level 4 in SAT-UK tests upon leaving primary education. In order to explore whether poor comprehenders show poor educational attainment at this point, the percentage of children in this group obtaining a level 4 across reading, writing, science and maths tests was compared to (1) the control group, and (2) national data. National data (UK Department for Education, 2012a) refer to children in England completing SAT-UK tests during the same year in which the present participants completed these tests (total *n *≈ 584,500). **Figure 3** illustrates the percentage of children in the poor comprehender group, control group and nationally who achieved a level 4 or above in reading (10 poor comprehenders: 67%, 15 controls: 100%, national data: 84%), writing (9 poor comprehenders: 60%, 15 controls: 100%, national data: 67%), science (12 poor comprehenders: 80%, 15 controls: 100%, national data: 88%), and maths (11 poor comprehenders: 73%, 12 controls: 80%, national data: 77%).

All participants in the control group performed at or above a level 4 in reading, writing, and science (but not maths). In each subject however, a lower number of poor comprehenders achieved a level 4 or above in comparison to controls, and this difference was most marked for the reading and writing tests. Fisher’s exact tests (all 2-tailed) showed that there was a significant association between comprehension group (poor comprehenders vs. controls) and attainment (below level 4 vs. level 4 or higher) for reading (*p* = 0.04) and writing (*p *= 0.02), but not for science (*p* = 0.22) or maths (*p* = 1.00). When comparing the poor comprehender group to national data, a lower percentage of poor comprehenders achieved a level 4 or above across all subjects; Fisher’s exact tests revealed that the association between group (poor comprehender vs. national) and attainment (below level 4 vs. level 4 or higher) was significant for reading (*p* = 0.01) but not writing (*p* = 0.39), science (*p* = 0.17) or maths (*p* = 0.50). Finally, a higher percentage of the control group achieved a level 4 or above in comparison to the national data; Fisher’s exact tests revealed that the association between group (control vs. national) and attainment (below level 4 vs. level 4 or higher) was significant for writing (*p* = 0.02) but not reading (*p* = 0.38), science (*p* = 0.38) or maths (*p* = 0.74).

### EDUCATIONAL ATTAINMENT AT 16 YEARS (TIME 3)

As mentioned above, data on educational attainment at 16 years were only available for 21 of the 30 participants. One-way ANOVA and Fisher’s exact tests were conducted as appropriate, confirming that there were no systematic differences between those participants who were retained within the sample and those who were not on age, gender, nonverbal reasoning, reading, vocabulary and SAT-UK performance (all *p*s > 0.05). **Table 2** summarizes means and standard deviations for poor comprehender and control groups on GCSE exams (or equivalent), which occur at the end of compulsory schooling in the UK. **Table 2** indicates the total number of qualifications taken, total points obtained and average points obtained. To mirror the SAT-UK test scores reported above, we also present average points obtained in English, maths, and science subjects (note that a maths points score was not available for one participant in the poor comprehender group). Compared to the controls, there were clear trends for the poor comprehenders to take fewer subjects at GCSE, obtain fewer points overall and perform less well on English. However, these group differences did not reach statistical significance (although effect sizes were small to moderate, see **Table 2**).

**Table 2:** Summary of education outcomes at Time 3 (age 16 years).

Measure	Poor comprehenders (*n* = 11)		Controls (*n* = 10)		Group comparisons		
	*M*	*SD*	*M*	*SD*	*F*	*p*	Cohen’s *d*
Number of qualifications	7.95	2.77	9.35	2.21	1.61	0.22	0.56
Total points	362.73	186.66	406.90	151.88	0.35	0.56	0.26
Average points	44.66	14.21	42.73	12.22	0.11	0.74	0.15
Average English points	37.82	8.81	41.40	16.84	0.38	0.54	0.28
Average Science points	41.11	12.11	42.70	9.79	0.11	0.75	0.15
Average Maths points^1^	40.87	13.64	41.27	14.78	0.00	0.95	0.03

*^1^One participant in the poor comprehender group did not obtain a GCSE maths score therefore the mean for the poor comprehender group is based on 10 participants only.*

In a final set of analyses, we considered two key government targets (for details, see Materials and Methods section above). When the government report on attainment at the end of compulsory education, key indices are whether children obtain five or more GCSEs (or equivalent) at level 2, and whether they make “expected progress” between SAT-UK and GCSE examinations in English and maths. In our sample, 6/11 children in the poor comprehender group (55%) achieved five or more level 2 grades (or equivalent), compared to 7/10 children in the control group (70%). A Fisher’s exact test revealed that there was no significant association between comprehension group (poor comprehenders vs. controls) and whether or not participants achieved five or more level 2 grades (*p* = 0.66). We then compared the percentage of pupils in each comprehension group who obtained five or more level 2 GCSE grades (or equivalent) to the national percentage of pupils in England (83%; total *n *≈ 561,300) for the same calendar year (UK Department for Education, 2012b). For the comparison with the poor comprehender group, the Fisher’s exact test indicated that there was a significant association between group (poor comprehender vs. national) and attainment (five level 2 vs. not; *p *= 0.03). For the comparison between the controls and national data, this association was not significant (*p* = 0.39).

By ascertaining whether children made expected progress, it is possible to tap into the relationship between SAT-UK and GCSE performance. In English, 7/11 poor comprehenders (64%) and 7/10 controls (70%) made expected progress; in maths, 7/10 poor comprehenders (70%, one poor comprehender did not take maths GCSE) and 7/10 controls made expected progress. The same seven controls made expected progress across English and maths subjects. For poor comprehenders, there was almost complete overlap across subjects, with the exception of one poor comprehender making expected progress in English and not taking a maths GCSE (it is unclear why as English, maths and science are compulsory), and another poor comprehender making expected progress in maths but not English. A Fisher’s exact test revealed that there was no significant association between comprehension group (poor comprehenders vs. controls) and whether or not participants made expected progress for English (*p* = 1.00) or maths (*p* = 1.00). When these groups were compared to the number of pupils nationally who made expected progress in English (69%; total *n *≈ 522,782) and maths (70%; *n *≈ 522,709) over the same time frame, there were no significant associations for either poor comprehender (English: *p *= 0.75; maths: *p *= 1.00) or control (English: *p *= 1.00; maths: *p *= 1.00) groups.

## DISCUSSION

Despite a wealth of research investigating cognitive and linguistic skills in poor comprehenders in Italy, the UK and the US (e.g., Catts et al., 2006; Carretti et al., 2009; Nation et al., 2010), and the likely constraint that reading comprehension difficulties will place on educational progress, research on educational attainment was previously restricted to just one study, conducted in the UK with 11-year-old children (Cain and Oakhill, 2006). In the present study, data on national educational attainment tests in the UK were collected in order to explore whether poor comprehenders first recruited at age 9 years show poor educational outcomes at the end of primary school (age 11 years) and at the end of compulsory schooling (age 16 years). Data collected at ages 9 and 11 years also enabled investigation of reading and oral vocabulary development.

At 11 years, approximately a third of poor comprehenders failed to meet government targets on reading and writing tests and there was clear evidence for low achievement in reading compared to the national data set. Poor comprehenders showed lower scores on reading and writing tests compared to controls without a history of reading comprehension difficulties, despite groups being closely matched for age, general cognitive ability and decoding skill. Therefore, our findings point to a link between reading comprehension (and oral vocabulary) difficulties and poor educational attainment that cannot be explained by decoding or general cognitive ability. In the main, our study replicates Cain and Oakhill (2006), who showed differences in educational attainment on these tests between poor comprehenders and a similar control group. However, in contrast to Cain and Oakhill’s (2011) study, differences between poor comprehenders and controls were restricted to English tests (i.e., group differences on reading and writing but not maths and science). Given marked heterogeneity in the profiles of children described as poor comprehenders (Nation et al., 2002; Cain and Oakhill, 2006; Floyd et al., 2006), differences between studies are perhaps to be expected.

At 16 years, evidence for low educational attainment in poor comprehenders was less clear. When poor comprehenders were compared to controls, there were no significant differences on any of the indices of achievement, although on almost all measures, poor comprehenders performed less well than controls. It is worth noting, however, that nearly one in two of our poor comprehenders failed to achieve five GCSEs at A* to C, compared to approximately one in six nationally. Taken together with findings from age 11 years, our study indicates that poor comprehenders are at risk of educational failure at the end of primary school, and may also be at a disadvantage at the end of compulsory education.

Findings on attainment at 16 years should be treated with caution as data were only available for a subsample of poor comprehenders (11/15) and controls (10/15). Given the small sample size, and therefore limited power, it is perhaps not surprising that differences between poor comprehender and control groups were not statistically significant. In addition, we were not able to collect individual data on reading and other aspects of cognitive functioning at age 16 years, thus the reading (and oral vocabulary) status of participants at this point is unknown. Also unknown is whether any children had support during examinations (e.g., extra time, scribe). Nonetheless, to our knowledge, we provide the first study investigating educational attainment in poor comprehenders at the end of compulsory education. Further, our finding that children with a history of reading comprehension difficulties are less likely than pupils nationally to obtain five GCSEs at A* to C warrants further investigation: this is an index that is widely used by UK educational institutions and employers to make recruitment decisions and failing to obtain five GCSEs at A* to C is associated with greater risk of falling into the category of school leavers who are “Not in Employment, Education or Training” (NEET; UK Department for Education, 2010).

Alongside collecting data on educational attainment in poor comprehenders, we also tracked reading and vocabulary longitudinally. Reading and vocabulary measures were administered when poor comprehenders were identified at age 9 years and after a 2-year lag at age 11 years. Raw reading comprehension scores for poor comprehenders and controls increased significantly over time but this increase was more marked for the poor comprehenders (see **Figure 1**). For poor comprehenders, reading comprehension standard scores showed stability; with one or two exceptions, they showed little change over time. Controls’ standard scores declined indicating that their improvements were not commensurate with the age-related differences reported for the test’s normative sample. This is a surprising finding, and one that warrants further attention. Importantly though, the group difference in reading comprehension (raw and standard scores) maintained over time and the gap between low and high ability groups did not appear to widen (i.e., a Matthew effect), consistent with previous research (Scarborough and Parker, 2003; Cain and Oakhill, 2006, 2011; Elwér et al., 2013).

Mean oral vocabulary scores (raw, standard) for poor comprehenders were significantly lower than mean scores for controls at both Time 1 and Time 2. Over time, scores for the two groups showed parallel growth, with raw scores increasing and mean standard scores not changing significantly between ages 9 and 11 years (see **Figure 1**). Therefore, and in contrast to Cain and Oakhill (2011), we did not find evidence for Matthew effects in the oral vocabulary knowledge of poor comprehenders. Rather, they demonstrated poorer oral vocabulary knowledge than controls at Time 1, and this group difference was maintained (but did not increase) over time (cf. Scarborough and Parker, 2003). Given the discrepancy between our findings and those of Cain and Oakhill, it is worth noting that Cain and Oakhill (2011) identified their poor comprehenders using different criteria. In addition, markedly different measures of oral vocabulary were used across the studies. Cain and Oakhill (2011) used a receptive measure, with scores determined by the breadth of oral vocabulary knowledge (i.e., how many words a child knows) whereas our expressive measure was more sensitive to the incremental nature of oral vocabulary, with scores capturing depth as well as breadth of knowledge. In order to investigate further whether poor comprehenders are at risk of Matthew effects for vocabulary, future research should aim to administer multiple measures of oral vocabulary, indexing vocabulary knowledge in relation to breadth, depth, and flexibility (e.g., understanding multiple meanings) and how this knowledge can be used.

In conclusion, we have replicated findings that poor comprehenders are at risk for poor educational attainment at the end of primary school (Cain and Oakhill, 2006). At this point, poor comprehenders were more likely to perform poorly, and fail to reach government targets, than controls and the national sample on literacy tests. We also extended this by providing preliminary evidence that some poor comprehenders show low educational outcomes at the end of compulsory education (16 years); compared to the national sample, poor comprehenders were less likely to obtain five or more A* to C GCSE grades (or equivalent). These findings indicate that more research on educational attainment in poor comprehenders is warranted. A key outstanding empirical question is *why* some poor comprehenders perform poorly in national school assessments. The complexity of these assessments means that there are a large number of factors that could constrain performance and given the heterogeneity of poor comprehenders, different factors could explain poor performance for different individuals. Further research is needed that tracks educational attainment in a more systematic and detailed way, and with large enough groups in order to investigate different trajectories. For instance, it would be of value to determine which factors (e.g., reading comprehension level, oral language abilities, ability to learn from reading, etc.) predict the likelihood that poor comprehenders will go on to perform poorly at school. A further complication for interpreting our findings is that SAT-UK and GCSE assessments are not directly comparable. For example, SAT-UK English tests measure reading ability directly whereas GCSE English assessments do not. Thus, there may be different reasons for poor performance at different educational stages. Future research that analyses the content of the tests taken could shed light on this issue, and probe the implications of this work for curriculum development and education in the UK. Finally, given that the extant literature comprises just two UK studies, future studies should aim to investigate links between poor reading comprehension and educational attainment in children outside of the UK. Difficulties with reading comprehension in childhood do not seem to guarantee poor educational outcomes and clearly there are a number of other variables that will influence national assessment scores. Taken together though, our findings do point to a link between reading comprehension difficulties in mid to late childhood and poor educational attainment further down the line.

## AUTHOR CONTRIBUTIONS

For the present study, Jessie Ricketts led in relation to study design, data collection, data analysis and the interpretation of the resulting dataset. Rachael Sperring contributed to data collection, data analysis and interpretation, and Kate Nation to study design, data analysis and interpretation. All authors contributed to manuscript preparation, with Jessie Ricketts taking the lead. All authors have approved the manuscript and take responsibility for all aspects of the work.

## Conflict of Interest Statement

The authors declare that the research was conducted in the absence of any commercial or financial relationships that could be construed as a potential conflict of interest.

## Footnotes

1 Note that since this study was conducted the age at which compulsory schooling ends in the UK has been raised from 16 to 17 years.
